# Associations Between Eight Earth Observation‐Derived Climate Variables and Enteropathogen Infection: An Independent Participant Data Meta‐Analysis of Surveillance Studies With Broad Spectrum Nucleic Acid Diagnostics

**DOI:** 10.1029/2021GH000452

**Published:** 2022-01-01

**Authors:** Josh M. Colston, Benjamin F. Zaitchik, Hamada S. Badr, Eleanor Burnett, Syed Asad Ali, Ajit Rayamajhi, Syed M. Satter, Daniel Eibach, Ralf Krumkamp, Jürgen May, Roma Chilengi, Leigh M. Howard, Samba O. Sow, M. Jahangir Hossain, Debasish Saha, M. Imran Nisar, Anita K. M. Zaidi, Suman Kanungo, Inácio Mandomando, Abu S. G. Faruque, Karen L. Kotloff, Myron M. Levine, Robert F. Breiman, Richard Omore, Nicola Page, James A. Platts‐Mills, Ulla Ashorn, Yue‐Mei Fan, Prakash Sunder Shrestha, Tahmeed Ahmed, Estomih Mduma, Pablo Penatero Yori, Zulfiqar Bhutta, Pascal Bessong, Maribel P. Olortegui, Aldo A. M. Lima, Gagandeep Kang, Jean Humphrey, Andrew J. Prendergast, Robert Ntozini, Kazuhisa Okada, Warawan Wongboot, James Gaensbauer, Mario T. Melgar, Tuula Pelkonen, Cesar Mavacala Freitas, Margaret N. Kosek

**Affiliations:** ^1^ Division of Infectious Diseases and International Health University of Virginia School of Medicine Charlottesville VA USA; ^2^ Department of Earth and Planetary Sciences Johns Hopkins Krieger School of Arts and Sciences Baltimore MA USA; ^3^ Division of Viral Diseases US Centers for Disease Control and Prevention Atlanta GA USA; ^4^ Department of Pediatrics and Child Health Aga Khan University Karachi Pakistan; ^5^ Department of Pediatrics National Academy of Medical Sciences Kanti Children's Hospital Kathmandu Nepal; ^6^ Division of Infectious Diseases Programme for Emerging Infections International Centre for Diarrhoeal Disease Research, Bangladesh (ICDDR,B) Dhaka Bangladesh; ^7^ Department of Infectious Disease Epidemiology Bernhard Nocht Institute for Tropical Medicine (BNITM) Hamburg Germany; ^8^ Centre for Infectious Disease Research in Zambia Lusaka Zambia; ^9^ Department of Pediatrics Vanderbilt University Medical Center Nashville TN USA; ^10^ Centre pour le Développement des Vaccins, Mali Bamako Mali; ^11^ Medical Research Council Unit The Gambia at the London School of Hygiene & Tropical Medicine Banjul The Gambia; ^12^ Epidemiology and Health Economics GSK Vaccine Wavre Belgium; ^13^ Department of Pediatrics and Child Health The Aga Khan University Karachi Pakistan; ^14^ National Institute of Cholera and Enteric Diseases Kolkata India; ^15^ Centro de Investigação em Saúde de Manhiça Manhiça Mozambique; ^16^ Centre for Nutrition & Food Security International Centre for Diarrhoeal Disease Research, Bangladesh (ICDDR,B) Dhaka Bangladesh; ^17^ Department of Pediatrics University of Maryland School of Medicine Baltimore MD USA; ^18^ Departments of Medicine and Pediatrics Center for Vaccine Development and Global Health University of Maryland School of Medicine Baltimore MD USA; ^19^ Global Health Rollins School of Public Health Emory University Atlanta GA USA; ^20^ Kenya Medical Research Institute Center for Global Health Research Kisumu Kenya; ^21^ Centre for Enteric Diseases National Institute for Communicable Diseases Pretoria South Africa; ^22^ Center for Child, Adolescent and Maternal Health Research Tampere University Tampere Finland; ^23^ Department of Child Health Institute of Medicine of Tribhuvan University Kirtipur Nepal; ^24^ Division of Nutrition and Clinical Services International Centre for Diarrhoeal Disease Research, Bangladesh (ICDDR,B) Dhaka Bangladesh; ^25^ Haydom Global Health Institute Haydom Tanzania; ^26^ HIV/AIDS & Global Health Research Programme University of Venda Thohoyandou South Africa; ^27^ Asociacion Benefica PRISMA Iquitos Peru; ^28^ Department of Physiology and Pharmacology Faculty of Medicine Federal University of Ceará Fortaleza Brazil; ^29^ Department of Gastrointestinal Sciences Christian Medical College Vellore India; ^30^ Department of International Health Johns Hopkins Bloomberg School of Public Health Baltimore MA USA; ^31^ Centre for Genomics and Child Health Blizard Institute Queen Mary University of London London UK; ^32^ Zvitambo Institute for Maternal and Child Health Research Harare Zimbabwe; ^33^ Research Institute for Microbial Diseases Osaka University Osaka Japan; ^34^ Department of Medical Sciences National Institute of Health Nonthaburi Thailand; ^35^ Department of Epidemiology Colorado School of Public Health Center for Global Health Aurora CO USA; ^36^ Pediatric Infectious Diseases Hospital Roosevelt Guatemala City Guatemala; ^37^ Children's Hospital Helsinki University Central Hospital Helsinki Finland; ^38^ Hospital Pediátrico David Bernardino Luanda Angola; ^39^ Division of Infectious Diseases and International Health and Public Health Sciences University of Virginia School of Medicine Charlottesville VA USA

**Keywords:** diarrheal disease, infectious diseases, weather, climate, hydrometeorology, pediatrics

## Abstract

Diarrheal disease, still a major cause of childhood illness, is caused by numerous, diverse infectious microorganisms, which are differentially sensitive to environmental conditions. Enteropathogen‐specific impacts of climate remain underexplored. Results from 15 studies that diagnosed enteropathogens in 64,788 stool samples from 20,760 children in 19 countries were combined. Infection status for 10 common enteropathogens—adenovirus, astrovirus, norovirus, rotavirus, sapovirus, *Campylobacter*, ETEC, *Shigella*, *Cryptosporidium* and *Giardia*—was matched by date with hydrometeorological variables from a global Earth observation dataset—precipitation and runoff volume, humidity, soil moisture, solar radiation, air pressure, temperature, and wind speed. Models were fitted for each pathogen, accounting for lags, nonlinearity, confounders, and threshold effects. Different variables showed complex, non‐linear associations with infection risk varying in magnitude and direction depending on pathogen species. Rotavirus infection decreased markedly following increasing 7‐day average temperatures—a relative risk of 0.76 (95% confidence interval: 0.69–0.85) above 28°C—while ETEC risk increased by almost half, 1.43 (1.36–1.50), in the 20–35°C range. Risk for all pathogens was highest following soil moistures in the upper range. Humidity was associated with increases in bacterial infections and decreases in most viral infections. Several virus species' risk increased following lower‐than‐average rainfall, while rotavirus and ETEC increased with heavier runoff. Temperature, soil moisture, and humidity are particularly influential parameters across all enteropathogens, likely impacting pathogen survival outside the host. Precipitation and runoff have divergent associations with different enteric viruses. These effects may engender shifts in the relative burden of diarrhea‐causing agents as the global climate changes.

## Key Points

- Many pathogens cause diarrhea in children and are affected in different ways by weather conditions like rainfall, temperature, and humidity
- Diarrhea‐causing bacteria infections increase in warm, humid weather and when soil is moist, though ETEC is sensitive to rainfall extremes
- High humidity decreases prevalence of several viruses, though only rotavirus increases in cold weather and following heavy surface runoff

## 1 Introduction

In spite of impressive decreases in global diarrheal disease burden this century, the syndrome remains a leading cause of childhood mortality and disease (Kotloff et al., 2013; Reiner et al., 2020) thought to have accounted for some 533,800 deaths in children under 5 years of age in 2017 (Roth et al., 2018) and with health and economic impacts that last throughout the life course (Dewey & Begum, 2011; McGovern et al., 2017). Most diarrhea is infectious in etiology, caused by numerous species of fecal‐orally transmitted viral, bacterial and protozoal microorganisms that vary in the extent to which environmental and atmospheric conditions facilitate or constrain their survival and dispersal (Chao et al., 2019; J. M. Colston et al., 2019; Platts‐Mills et al., 2018). This sensitivity to time‐varying hydrometeorological factors, now widely documented both for individual diarrhea‐causing pathogens (Brunn et al., 2018; Djennad et al., 2019; L.‐P. Wang et al., 2021; P. Wang et al., 2018) and for all‐cause diarrheal outcomes (Aik et al., 2020; Horn et al., 2018; Wangdi & Clements, 2017), has led to fears that climate change might undermine progress in reducing global childhood diarrheal disease incidence and deaths (K. Levy et al., 2016). Other researchers have projected that the burden of diarrhea of viral etiology may actually decrease in some locations due to shifting weather patterns (Onozuka et al., 2019). The lack of clarity surrounding the associations between meteorological exposures such as temperature, rainfall and humidity and the prevalence and transmission of individual enteropathogen species has stymied efforts to generate actionable projections of future disease burden trends under plausible climate change scenarios (Kolstad & Johansson, 2011; World Health Organization, 2014b).

Over the past decade considerable progress has been made in elucidating these relationships due to improved accessibility, accuracy, and resolution of climatological data on the one hand (J. M. Colston, Ahmed, Mahopo, et al., 2018) and sensitivity and affordability of broad‐based tools for differential diagnosis of diarrhea‐causing agents on the other (Brown & Cumming, 2019). Concurrent advances in statistical methods are now able to account for complex non‐linear, time‐lagged and highly seasonally varying associations (Alsova et al., 2019; J. M. Colston et al., 2019), and analysis of data pooled from multiple sites and studies has been demonstrated to offer insights into the general epidemiology of enteric pathogens that might not be apparent from considering just a single location (Andersson et al., 2018; J. M. Colston et al., 2020; Hasso‐Agopsowicz et al., 2019). Analyses of time‐series data from particular cities, regions or countries have identified significant associations between individual climatological parameters—such as relative humidity in Singapore (Aik et al., 2020), temperature in Bhutan (Wangdi & Clements, 2017) and precipitation in Mozambique (Horn et al., 2018)—and diarrheal outcomes. Others have focused on single pathogen species (Brunn et al., 2018; Djennad et al., 2019; Hasan et al., 2018; Ureña‐Castro et al., 2019) or compared multiple species within the same taxon (Park et al., 2018; P. Wang et al., 2018). However, to draw broad, generalizable conclusions about the impact of weather on enteropathogens, it is necessary to combine data from locations that are representative of different climate zones. Initial efforts to do this for rotavirus in the 8‐site MAL‐ED study (J. M. Colston et al., 2019), for multiple pathogens in the 7‐site GEMS study (Chao et al., 2019) and across seven ecological regions of China (L.‐P. Wang et al., 2021) illustrate the value and potential of such approaches.

The objective of this Independent Participant Data Meta‐Analysis (IPD‐MA) was to pool data from comparable studies in multiple locations across diverse geographical areas and climate zones and match them with coincident Earth observation‐derived data to model the associations between eight hydrometeorological exposures (precipitation, runoff volume, humidity, soil moisture, solar radiation, air pressure, temperature, and wind speed) and infection status for 10 common, high‐burden enteric pathogens ascertained in young children (adenovirus, astrovirus, norovirus, rotavirus, sapovirus, *Campylobacter*, ETEC, *Shigella*, *Cryptosporidium*, and *Giardia*). The research question to be assessed was whether different combinations of rainfall, ambient temperature, atmospheric humidity and pressure and other parameters impact the risk of enteric infections independently of each other and of seasonality, and at magnitudes and in directions that differ by pathogen species, which are differentially resistant to environmental conditions such as dryness, ultraviolet light, and in their probability of transmission via aerosol (J. M. Colston et al., 2019; Fernstrom & Goldblatt, 2013; Ijaz et al., 1985; Jones & Brosseau, 2015). Understanding these associations may inform health care policy and planning including prioritization of target populations for existing vaccines (rotavirus) or the further development of promising candidates (*Shigella*, enterotoxigenic *E coli* [ETEC], and multicomponent viral vaccines).

## 2 Methods

### 2.1 Sources of Pathogen Data

**Figure 1:**
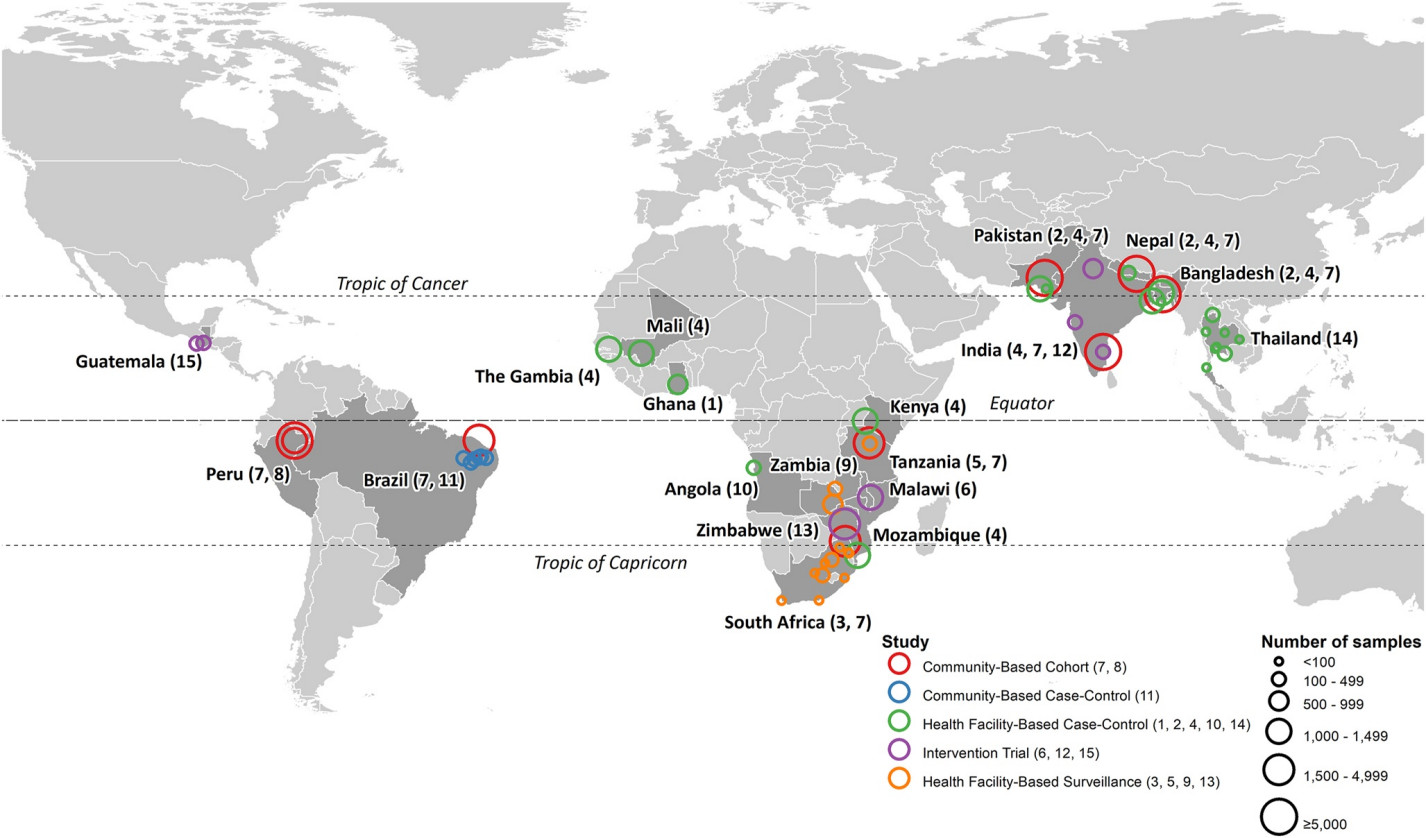
Locations of the sites of the studies contributing data to the analysis, and number of samples from each site included in the analysis.

This analysis used an IPD‐MA framework in which raw, individual participant‐level data was pooled from studies that used molecular diagnostics to identify multiple enteric pathogens in stool samples collected from children aged under 5 years in Low‐ and Middle‐Income Countries (LMICs). The IPD‐MA design is considered the gold standard in systematic reviews, offering numerous advantages over aggregate data meta‐analyses, and a greater potential for generalizable inferences compared with individual studies (Chen & Benedetti, 2017; Dewidar et al., 2021). In this IPD‐MA, studies which tested for six or more of 10 target pathogens were identified through exploratory literature review and professional networks and investigators from eligible studies were contacted with requests for access to their data. Study‐specific datasets were combined into a central database with a standardized format and list of variables and analyses were conducted on the pooled data. Table 1 summarizes key features of studies that contributed data (throughout this article, studies are referred to by the number in the leftmost column of the table), while Figure 1 shows the locations and number of samples included from each of the studies' sites. 15 studies contributed data from 19 countries with a range of latitudes spanning the entirety of the tropics and sub‐tropics, including locations in Central and South America (studies 7, 8, 11, and 15), Sub‐Saharan Africa (1, 3, 4, 5, 6, 7, 9, 10, and 13) and South and Southeast Asia (2, 4, 7, 12, and 14). Study designs varied and included numerous case‐control designs (1, 2, 4, 10, 11, and 14) in which a single sample was collected from active cases of diarrhea and matched to those from asymptomatic controls. Some other studies used health facility‐based surveillance (3, 5, and 9) and so only collected samples from subjects during an ongoing diarrhea episode of a severity necessitating care‐seeking. Still others had community‐based, longitudinal designs (6, 7, 8, and 13), mostly collecting repeated asymptomatic samples from the same subjects along with a small number of samples from uncomplicated diarrhea episodes as they occurred. Several studies were carried out at multiple sites, including in different countries (2, 4, and 7), while some sites were included in more than one study (7 and 12 in Vellore, India; 5 and 7 in Haydom, Tanzania; 7 and 8 in Loreto, Peru). This meant that the pooled database had a complex, hierarchical structure with samples nested in subjects, which in turn were nested in sites, but sites were not uniformly nested within studies and vice versa.

**Table 1:** Features of Studies That Contributed Data to This Analysis

Study		Sites	Design	Inclusion criteria	Sample collection schedule	Follow‐up period	Number of included samples (subjects)	Reference
1.	Agogo Presbyterian Hospital	Asante Akim North Municipality, Ghana	Health facility‐based unmatched case‐control study	Watery/bloody diarrhea cases age <6 years, hospital controls	One per subject collected within 24 hr of enrollment	2007–2008	646 (606)	Eibach et al. (2016)
2.	Asian Intussusception Surveillance Network (AISN)	Various locations in Bangladesh, Nepal, Pakistan	Health facility‐based matched case‐control study	Intussusception cases age <2 years, matched hospital controls	One per subject collected within 48 hr of enrollment	2015–2017	206 (206)	Burnett et al. (2020)
3.	Diarrheal Sentinel Surveillance Program (DSSP)	Various locations in South Africa	Health facility‐based surveillance study	Acute diarrhea patients aged <5 years	One per subject collected within 48 hr of admission	2009–2017	689 (689)	Page et al. (2018)
4.	Global Enteric Multicenter Study (GEMS)	Mirzapur, Bangladesh; Basse, The Gambia; Kolkata, India; Nyanza, Kenya; Bamako, Mali; Manhiça, Mozambique; Karachi, Pakistan	Health facility‐based matched case‐control study	Moderate‐to‐severe diarrhea cases aged <5 years, matched community controls	1 per subject collected at enrollment	2007–2011	11,276 (10,769)	Kotloff et al. (2012)
5.	Haydom Lutheran Hospital	Haydom, Tanzania	Health facility‐based surveillance study	Gastroenteritis/diarrhea patients aged <5 years	One per subject collected within 48 hr of enrollment	2014–2015	233 (233)	Platts‐Mills et al. (2017)
6.	iLiNS‐DYAD	Mangochi District, Malawi	Community‐based trial of a nutritional product	Children of women enrolled during pregnancy	Three asymptomatic samples per subject collected at 6, 18, and 30 months of age	2011–2014	1,835 (711)	Ashorn et al. (2015)
7.	Malnutrition and Enteric Disease Study (MAL‐ED)	Dhaka, Bangladesh; Fortaleza, Brazil; Vellore, India; Bhaktapur, Nepal; Naushahro Feroze, Pakistan; Loreto, Peru; Venda, South Africa; Haydom, Tanzania	Community‐based cohort study	Newborns with normal birth weight	Monthly from 0 to 24 months of age and upon caregiver reported diarrhea episode (of any severity)	2009–2014	41,327 (1,715)	MAL‐ED Network Investigators (2014)
8.	Novel Biomarkers of Environmental Enteropathy (NBEE)	Loreto, Peru	Community‐based cohort study	Children aged 3–18 months	15 per subjects between 1 and 30 days following enrollment	2018	1,075 (75)	Bill and Melinda Gates Foundation (2012)
9.	Program for Awareness and Elimination of Diarrhea (PAED)	Lusaka and Ndola, Zambia	Health facility‐based surveillance study	Moderate‐to‐severe diarrhea patients aged <5 years	One per subject collected at enrollment	2012–2013	1,379 (1,379)	Chisenga et al. (2018)
10.	Pediatric Hospital of Luanda	Luanda, Angola	Health facility‐based unmatched case‐control study	Diarrhea cases (any severity) aged <5 years, unmatched hospital controls	One sample per subject collected at enrollment	2013–2014	194 (194)	Pelkonen et al. (2018)
11.	RECODISA	Six cities in the semiarid region of Brazil (Cajazeiras, Crato, Ouricuri, Patos, Picos, and Sousa)	Community‐based matched case‐control study	Diarrhea cases (any severity) aged 2–36 months, matched community controls	One per subject collected at enrollment	2009–2012	1,200 (1,200)	Lima et al. (2019)
12.	Rotavac Trial, India	Three sites in India (Delhi, Pune, and Vellore)	Placebo‐controlled vaccine efficacy trial	Infants aged 6–7 weeks	Upon caregiver reported diarrhea episode (any severity)	2011–2013	1,271 (1,090)	Bhandari et al. (2014)
13.	Sanitation Hygiene Infant Nutrition Efficacy (SHINE) Trial	Midlands, Zimbabwe	Community‐based WASH trial	HIV‐unexposed children of women enrolled during pregnancy	1, 3, 6, and 12 months of age and upon caregiver reported diarrhea episode (any severity)	2013–2016	2,372 (1,046)	Humphrey et al. (2019)
14.	Thai Hospitals	Various locations in Thailand	Health facility‐based matched case‐control study	Acute diarrhea cases (any age), matched hospital controls	One per subject collected at enrollment	2016–2018	473 (473)	Okada et al. (2020)
15.	Urban and rural Guatemala	El Trifinio region, and Guatemala City, Guatemala	Health facility‐based trial of a nutritional product	Acute non‐bloody diarrhea patients aged 6–35 months	Two per subject collected at enrollment and at study day 31	2015–2016	585 (299)	Gaensbauer et al. (2019)

In pooling the data, the following information was retained from the study‐specific databases in addition to the diagnostic results: the date of sample collection; the subjects' age on that date; whether the sample was collected while the subject was asymptomatic or during a diarrheal episode; whether the subject was enrolled in a health facility‐ or community‐based study; and the country, study, and site in which the subject was recruited. In addition, samples were georeferenced to the latitude and longitude coordinates of the approximate location of the subjects' residence, based on information provided by the study investigators. At some study sites, coordinates of the subjects' household locations were available (55.8% of samples), otherwise, subjects were georeferenced to the approximate centroid of their neighborhood, village or district (32.0%) or, where such information was unavailable, the location of the health facility that recruited them (12.2%). In the case of health facilities, this was based on the assumptions that subjects would seek care from facilities that were closest to where they reside, that some error would be introduced from using facility locations as an approximation of the true residence location, but that this error would be small relative to that introduced by the coarse resolution of the climate data.

### 2.2 Outcome Variables

The outcomes of interest were infection status for 10 common enteric pathogens ascertained by quantitative or real‐time polymerase chain reaction (PCR) performed on diarrheal and asymptomatic stool samples and treated as binary variables (positive or negative). The pathogens were selected for being either highly endemic or responsible for high diarrheal disease morbidity in low resource regions of the Global South (Operario et al., 2017; Platts‐Mills et al., 2018), as well as to be representative of the three major taxa and having PCR results available across the majority of included studies. These included: adenovirus (enteric serotypes 40 and 41); astrovirus; norovirus (either genogroup); rotavirus; sapovirus; *Campylobacter* spp.; ETEC; *Shigella* spp./enteroinvasive *E coli* (EIEC) (PCR uses the same gene target for these two related pathogens); *Cryptosporidium* spp.; and *Giardia* spp. For consistency across studies, where cycle threshold values were available, a 35‐cycle cutoff was used to define positivity. To ensure that a single infection episode was not counted multiple times, *Campylobacter*‐ and norovirus‐positive results were excluded if they were collected within 30 days of a previous sample from the same subject that was positive for the same pathogen strain without being separated by an intermediate negative sample (since these two pathogens are prone to induce more persistent episodes and longer duration of shedding) (J. M. Colston et al., 2020). For all other pathogens, a 14‐day period was used, except for the two protozoa, for which positive results for the same species (*C. parvum* or *C. hominis*) or assemblage (*G. duodenalis* A or B) as a prior sample from the same subject were excluded unless separated by three negative results (J. M. Colston et al., 2020; McMurry et al., 2020).

### 2.3 Exposure Variables

The exposure variables of interest were a set of historical daily Earth observation‐ and model‐based re‐analysis‐derived estimates of hydrometeorological variables taken from version 2.1 of the Global Land Data Assimilation System (GLDAS) (Rodell et al., 2004). These data are produced by merging observation and model‐based analysis, have been described and evaluated elsewhere (J. M. Colston, Ahmed, Mahopo, et al., 2018; J. M. Colston et al., 2019) and offer numerous advantages over ground‐ and station‐based measurements, primarily geographic and temporal completeness, as well as including variables not routinely reported from weather stations. GLDAS data are provided in the form of gridded raster files with a horizontal resolution of 0.25 decimal degrees (approximately 27 km near the equator). Given this relatively coarse resolution, multiple georeferenced residence locations often fall within the same GLDAS grid square, so these locations were linked to their geographically nearest grid square centroid. A script was run to extract all variable values at those centroids from all gridded GLDAS files from 2005 to 2019, the 15‐year period that includes the follow‐up period for all included studies. 3‐hourly estimates were aggregated to daily averages, totals, or minimum and maximum daily values as appropriate. Eight hydrometeorological variables were included based on their demonstrated or hypothesized potential to influence transmission of enteric pathogen species (J. M. Colston, 2018; K. Levy et al., 2016; L.‐P. Wang et al., 2021): daily total precipitation and surface runoff volume (mm); relative humidity (%); soil moisture (%); solar radiation (W/m^2^); surface pressure (mbar, a proxy for storminess); average daily temperatures (°C, calculated from the daily minimum and maximum); and wind speed (m/s). The hydrometeorological data were matched with the stool sample results based on location (nearest GLDAS grid square centroid to subjects' residence) and date.

**Figure 2:**
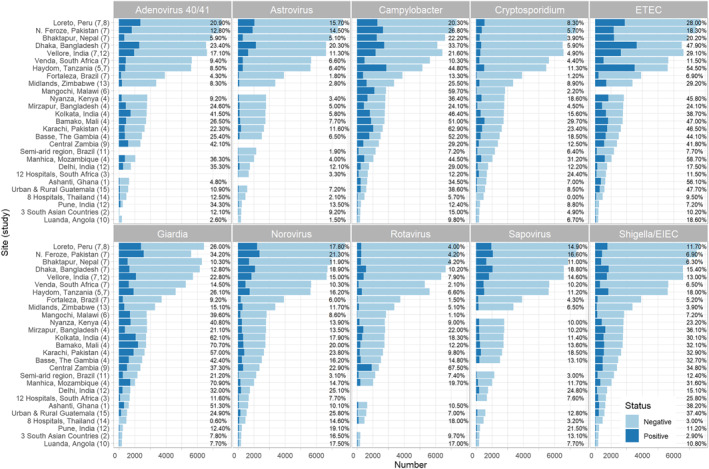
Number of stool samples testing positive and negative and positivity rate (%) for 10 enteropathogens by study site (ordered by total samples contributed with sites from several country‐specific and multi‐site studies combined).

Because of the assumed lagged effect of weather on pathogen outcomes (J. M. Colston et al., 2019; L.‐P. Wang et al., 2021) and the variability in incubation periods, which for most enteric agents are known to be short (Chai et al., 2019; R. M. Lee et al., 2013), hydrometeorological exposures were aggregated over a 7‐day moving time window from 3 to 9 days prior to the date of sample collection (*t*_−9_ to *t*_−3_, where *t*_*0*_ is the date of sample collection). For precipitation and surface runoff, the total volume over this period was summed; for all other exposures, the daily average value was calculated. Two of the hydrometeorological exposure variables, precipitation and surface pressure, were then standardized to their local distributions by recalculating each one as the deviation from its location‐specific mean value over the 15‐year period. For precipitation, this was to account for the distribution being highly positively skewed, while keeping the variable on a continuous scale, rather than categorizing or dichotomizing at certain percentiles. For surface pressure, which was narrowly distributed within each site, it was to reduce the between‐site relative to within‐site variability (Figure 2) (J. M. Colston et al., 2019). Because surface runoff was even more highly skewed than precipitation with a high proportion of zero values, numerous large outliers at the upper extreme and a maximum value of 214.7 mm, this variable was instead divided into three categories: no runoff (61.7%), light runoff (<5 mm, 32.8%) and heavy runoff (≥5 mm, 5.6%). The other exposure variables were centered at values close to their means. A minimal set of non‐hydrometeorological variables were included as covariates in the analysis:

1. **Age:** the subjects' age in continuous months at the time of sample collection (centered at 15 months).
2. **Sample type and study design:** Due to the inclusion of health facility‐based studies, diarrheal samples were overrepresented in the pooled database relative to the frequency of diarrhea occurrence in the general population, which could lead to overestimation of risk for pathogens strongly associated with diarrhea. To adjust for this, a binary categorical variable was included indicating whether the stool sample was collected while the subject was asymptomatic (the reference category) or during a diarrheal episode of any severity. A second binary variable was included indicating whether the sample was from a study with a community‐ (the reference category) or health facility‐based design and an interaction term was specified between these two variables, to account for the assumed differential pathogen positivity rates in diarrheal samples and particularly severe diarrhea for which facility care was sought.
3. **Country:** The country in which each sample was collected was included as a categorical variable with 15 categories and India (the country with the largest number of samples) as the reference category to control for the fixed effects of heterogeneity in the background levels of disease risk that may differ by country.
4. **Seasonality:** To adjust for potential residual confounding by unmeasured co‐seasonal factors, the sites were grouped into 4 broad epidemiological and climatological zones: Southern Asia (Bangladesh, India, Nepal, Pakistan, and Thailand—the reference category); Tropical Northern Hemisphere (the Gambia, Ghana, Guatemala, and Mali); Tropical Southern Hemisphere (Angola, Brazil, Kenya, Peru, and Tanzania); and Southern Africa (Malawi, Mozambique, South Africa, Zambia, and Zimbabwe). Terms for each seasonality zone's interactions with annual Fourier‐series sine and cosine function terms were included to allow for potential differences in zone‐specific timing and magnitude of seasonal patterns, but with the main effects of the zones suppressed (J. M. Colston, Ahmed, Soofi, et al., 2018; J. M. Colston et al., 2019). This approach was based on the assumption that the comparative seasonality of hydrometeorological variables and enteric disease transmission varies across different regions of the globe such that the timing and amplitude of annual peaks differ in the southern compared to the northern hemisphere (Bloom‐Feshbach et al., 2013; Martinez, 2018), in the tropics compared to the subtropics or poleward regions (Török et al., 1997) and in South and Southeast Asia compared to elsewhere due to the severity of the monsoon season there (Jagai et al., 2012). These assumptions are broadly consistent with the findings of both Colston and colleagues' analysis of rotavirus in MAL‐ED data (J. M. Colston, Ahmed, Soofi, et al., 2018) and Chao and colleagues' analysis of multiple pathogens in GEMS data (Chao et al., 2019).

### 2.4 Statistical Analysis

Samples were excluded if they were from subjects aged ≥5 years or with intussusception (a form of bowel obstruction) and if they were tested using a diagnostic method other than PCR. Two observations with outlier precipitation values (>300 m) were excluded. For each of the 10 pathogens, the probability of a stool sample being positive for that pathogen on a given day was estimated from the inverse logit‐transformed relative risks (RRs) by fitting modified Poisson models with robust variance estimation and clustering at the unit of the subject to the binary infection status outcomes using generalized linear models (GLMs) (J. M. Colston et al., 2019; Zou, 2004). All exposures and covariate variables were included together in models for each of the 10 pathogens (represented by model Equation S1 in Supporting Information S1). To account for potential non‐linear relationships between the continuous exposures and the pathogen outcomes, which have been documented for rotavirus (J. M. Colston et al., 2019; Hashizume et al., 2008), a multivariable spline model‐selection algorithm was applied to each model to select the best‐fitting number of degrees of freedom up to a maximum of four for each continuous variable (age plus the seven continuous hydrometeorological variables) (Royston & Sauerbrei, 2007). The modeled associations were visualized by plotting the probability of infection predicted by the models across the range of values of each continuous exposure variable or as dot‐and‐whisker plots for the surface runoff categories. To further assess relative effects, the GLMs were refitted for each pathogen‐exposure pairing, dichotomizing each continuous hydrometeorological exposure in turn at thresholds specific to that pairing and calculating RRs. This amounted to seven additional models per pathogen in each of which 1 hydrometeorological variable was dichotomized while the rest were kept as continuous. Each threshold was determined subjectively based on visualizations of the unadjusted (polynomial smooth plots) and adjusted (multivariable model predictions) associations (Figures 4 and 5). Multicollinearity was assessed by calculating variance inflation factors (VIFs) for all continuous model terms and verifying that all had a value of <10. As a sensitivity analysis, to verify that between‐site heterogeneity had been adequately accounted for using the fixed effects of county and diarrhea‐study design interaction, the analysis was repeated using mixed‐effects GLMs with random intercepts for subjects and study sites and the results compared to the main analysis. Mixed effects were not used in the main analysis since they yield attenuated effect estimates and large standard errors. In addition, the analysis was repeated after excluding diarrhea cases recruited through health facilities, to assess the sensitivity of the results to the inclusion of this type of sample. Analyses were carried out using Stata 16 (StataCorp, 2019), R 4.0.3 (R Core Team, 2020), and ArcMap 10.8 (ESRI, 2019).

## 3 Results

Results from 64,788 eligible stool samples from 20,760 children were analyzed. 75.1% of samples were collected when the child was asymptomatic, and 24.9% during a diarrheal episode (13.7% from subjects presenting to health facilities with diarrheal symptoms). Figure 2 shows positivity rates (%) and numbers of stool samples testing positive and negative for each of the 10 enteropathogens by study site. Where these statistics differ from those previously reported elsewhere, it is because multiple studies carried out at the same site have been combined, or because a small number of samples did not meet the criteria for inclusion in this analysis. The PCR targets used for each pathogen by each included study are listed in Table S1 of Supporting Information S1. All sites diagnosed *Campylobacter*, *Cryptosporidium*, *Giardia*, Norovirus, and *Shigella*/EIEC, while only one site in Malawi (6) did not test for ETEC. Three sites (6, 3, and 11) did not test for adenovirus 40/41, located respectively in Malawi, South Africa, and Brazil. This Brazilian study (11) instead tested for any adenovirus serogroup and, therefore, its results were excluded from the meta‐analysis. Three sites (6, 1, and 9) did not test for astrovirus or sapovirus, located respectively in Malawi, Ghana, and Zambia. Rotavirus PCR results were not available from two sites (3, 12), located in South Africa and in India. India's Rotavac trial (12) used other non‐comparable methods to diagnose rotavirus, which were excluded from the meta‐analysis.

**Figure 3:**
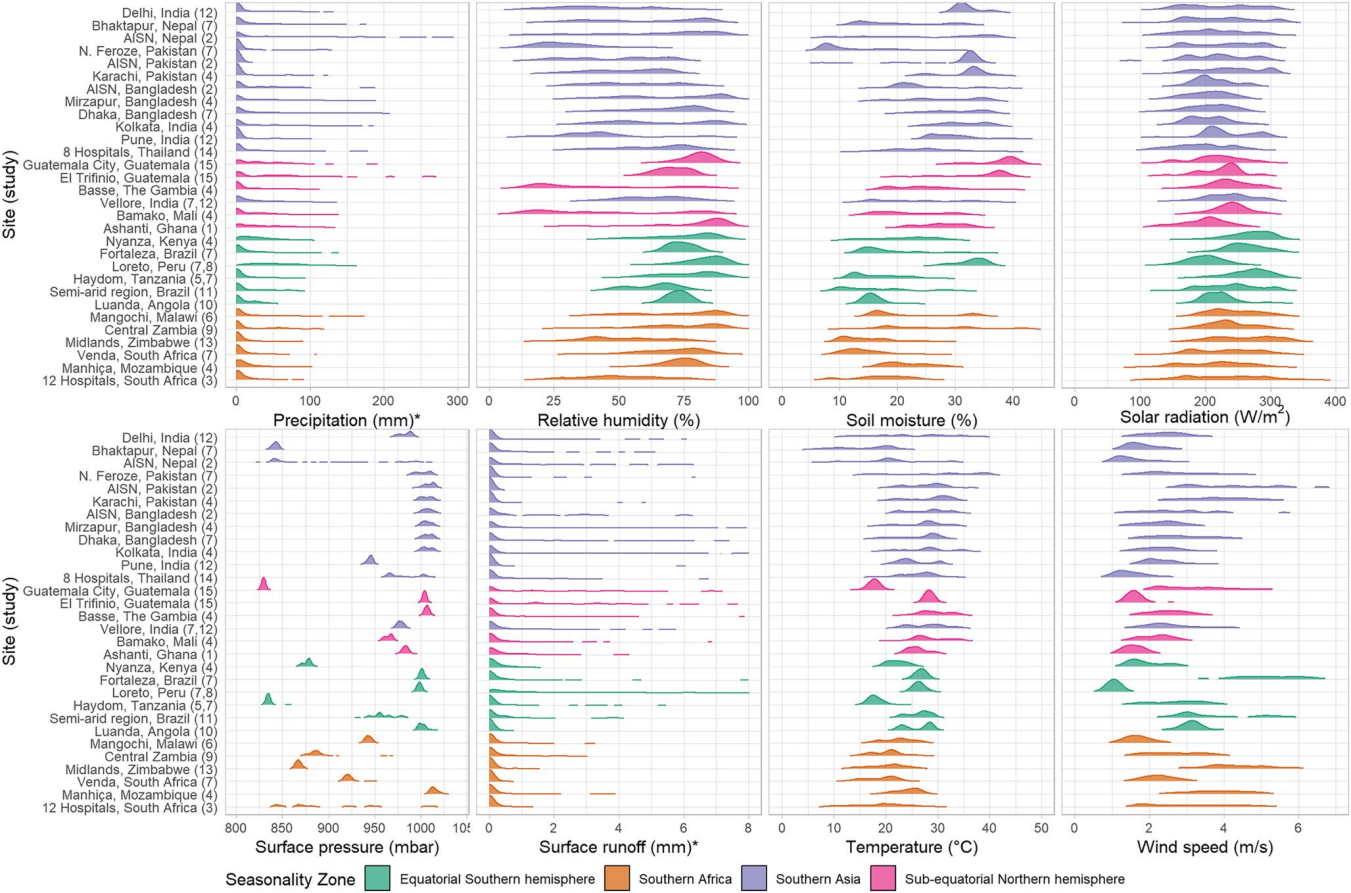
Density plots of the distribution of eight hydrometeorological variables in the contributing study sites, ordered by latitude from northernmost to southernmost. All are averaged over 7‐day windows lagged by three days from the stool samples (days *t*_−9_ to *t*_−3_ where *t*_0_ is the date of sample collection), except those indicated with * which are 7‐day totals. Outlier runoff values of >8 mm not shown.

Figure 3 shows the distributions of the daily values for the eight hydrometeorological exposure variables in each contributing study site prior to aggregating and categorizing runoff or standardizing precipitation and surface pressure. The standardized distributions of precipitation and surface pressure by site are shown in Figure S1 of Supporting Information S1. These data confirm that precipitation and runoff are uniformly highly skewed variables, though some evidence of variation in this skew was apparent for precipitation at the Ghana, Kenya, Peru and AISN Nepal sites, which showed relatively fewer dry days. Relative humidity had a fairly wide distribution in most sites with the exceptions of those in Latin America, Angola, and Mozambique, which showed higher levels of humidity with very few days in which it fell below 50%. Soil moisture tended to be more narrowly distributed within sites than relative humidity, particularly in Delhi, India; Luanda, Angola; and Loreto, Peru. The range of values for solar radiation was roughly consistent across sites, but there was evidence of a slight trend toward higher modal values at sites nearer the equator. Surface pressure was narrowly distributed within sites, with most sites having values clustered around approximately 1,000 mbar, but with high elevation sites (such as Bhaktapur, Nepal, Guatemala City, and Haydom, Tanzania) having much lower ranges below 850 mbar. The health facility‐based studies in Nepal (study 2.), Thailand (study 14.) and South Africa (study 3.) recruited subjects from multiple locations across their respective countries and this is reflected in the wider distributions of pressure values. Temperature exhibited a slight tendency to be more narrowly distributed in sites that were closer to the equator and in the Latin American sites (though in the case of the sites in Guatemala, this may be because that study 15. did not span a full year, so the data included may not be representative of the full temperature range at those locations). Wind speed showed considerable variability in its distribution across sites. For example, the Atlantic coastal city of Fortaleza, Brazil exhibited the windiest conditions (greater than 6 m/s), while the inland region of Loreto, Peru had very still conditions (below 2 m/s).

**Figure 4:**
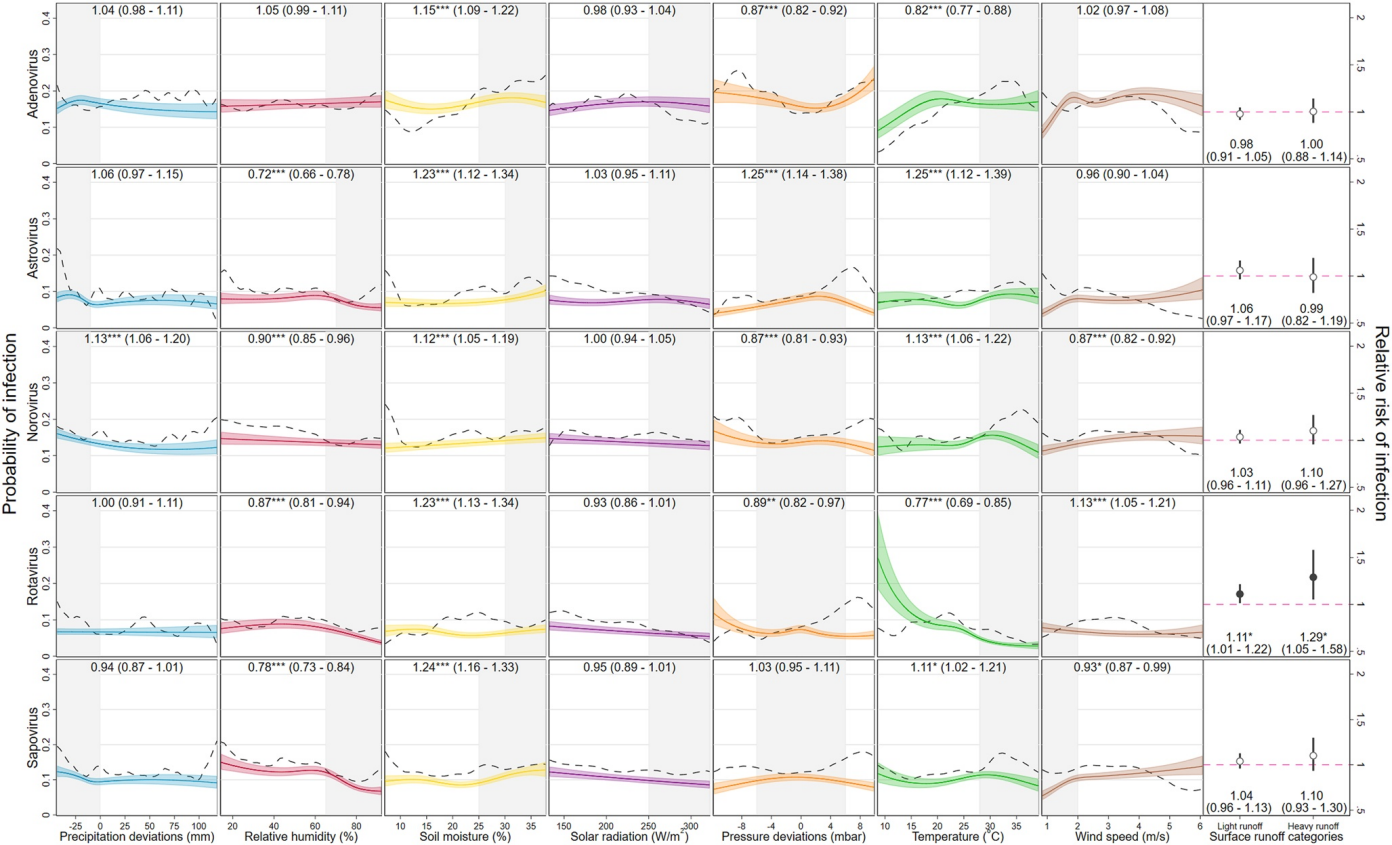
Associations between 8 hydrometeorological variables and infection status for five enteric viruses. Unadjusted effects (polynomial smoothed probability) shown by black, dashed lines; effects for continuous variables (predicted probability of infection from multivariable GLMs) shown by colored lines with 95% confidence intervals shown by shaded areas; relative risks of surface runoff categories shown by dot‐and‐whisker plots; panel captions are relative risks from otherwise identical GLMs in which each continuous variable in turn was dichotomized with the range shaded in gray as the comparison category (with 95% confidence intervals, ***p < 0.001, **p = 0.001–0.01, and *p = 0.01–0.05).

**Figure 5:**
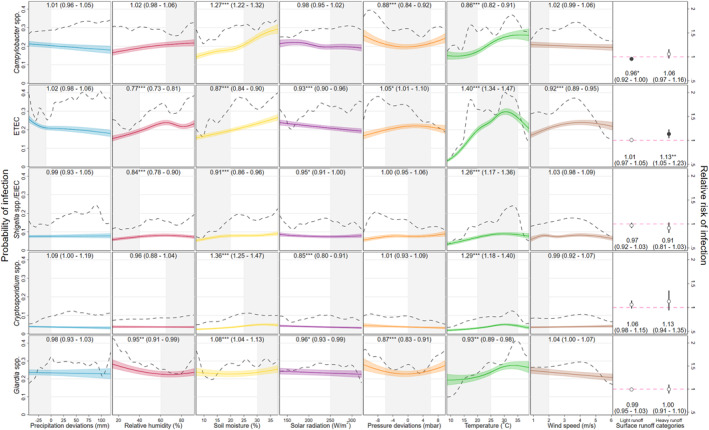
Associations between eight hydrometeorological variables and infection status for three enteric bacteria and two protozoa. Unadjusted effects (polynomial smoothed probability of infection) shown by black, dashed lines; effects for continuous variables (predicted probability of infection from multivariable GLMs) shown by colored lines with 95% confidence intervals shown by shaded areas; relative risks of surface runoff categories shown by dot‐and‐whisker plots; panel captions are relative risks from otherwise identical GLMs in which each continuous variable in turn was dichotomized with the range shaded in gray as the comparison category (with 95% confidence intervals, ***p < 0.001, **p = 0.001–0.01, and *p = 0.01–0.05).

Figures 4 and 5 plot the probabilities of infection predicted by the GLMs across the range of values for each of seven continuous hydrometeorological exposures with unadjusted (polynomial smooth plots) probabilities superimposed. RRs of infection are also plotted for the two comparison surface runoff categories and for each continuous variable after dichotomizing them at thresholds specific to each pathogen‐exposure pairing. Temperature showed statistically significant associations with all included pathogens, but with varying shapes and magnitudes. Among the viruses, rotavirus showed a strong inverse association with temperature, with predicted positivity ranging from around 25% at the coldest, to less than 5% at the warmest extremes and a 23% decreased relative risk above a threshold of 28°C (0.77 [0.69–0.85]). By contrast, the lowest predicted adenovirus positivity occurred at the coldest extreme, increasing to a peak at around 20°C, above which it plateaued, decreasing slightly with an 18% decreased risk (0.82 [0.77–0.88]) above a 28°C threshold. Astrovirus, norovirus and sapovirus exhibited more modest associations, with slight inverted U‐shaped associations in the upper range of the temperature distribution. For bacteria, the adjusted association between temperature and ETEC was the largest identified in this analysis, a skewed, inverted U‐shaped relationship, with predicted positivity lowest below 10°C (<5%) increasing steeply to a high of around 30% at around 32°C before declining slightly in the very hottest extremes and with a 40% (1.40 [1.34–1.47]) increased RR of positivity between 25 and 35°C. *Shigella* and the protozoa *Cryptosporidum* had similarly shaped associations with temperature as ETEC and with peaks at similar values but with much lower magnitudes, while the association with *Campylobacter* took on a more sinusoidal shape with a 14% (0.86 [0.82–0.91]) decreased risk of infection in the colder extreme of the distribution. The *Giardia* association took on a similar shape to *Campylobacter* but was of a lower magnitude (0.93 [0.89–0.96] below a 25°C threshold) and statistical significance.

Soil moisture showed small yet highly statistically significant associations with all 10 pathogens, and in each case higher probability of infection was predicted in the upper extreme of the distribution. The largest relative effect of soil moisture was with *Cryptosporidium*—a 36% increased risk above 25% moisture content (1.36 [1.25–1.47])—while large absolute effects were observed with *Campylobacter* and ETEC—a more than 10% point difference in infection probability at highest compared to lowest moisture.

Relative humidity showed low‐magnitude, statistically significant inverse associations with all enteric virus species with the exception of adenovirus, with which it demonstrated no discernable association. For astrovirus, rotavirus, and sapovirus, threshold effects were observed in the upper range of the humidity distribution, notably for astrovirus, for which there was a 28% (0.72 [0.66–0.78]) decreased relative risk above a 70% humidity threshold. The enteric bacteria all had roughly direct associations with relative humidity, though these were small in magnitude with the exception of ETEC, which showed a 23% (0.77 [0.73–0.81]) decrease risk below 40% humidity. *Cryptosporidium* showed no association and *Giardia* a slight inverse association with relative humidity. For adenovirus, *Campylobacter* and *Giardia*, lower risk of positivity was observed with pressure values within 6 mbar of the site‐specific mean, whereas for astrovirus, risk was 25% (1.25 [1.14–1.38]) higher in this range.

Although there was evidence that deviations in precipitation below the local average slightly increased infection risk for several pathogens (with the exceptions of rotavirus, *Shigella* and the two protozoa), only for norovirus did this translate into a statistically significant relative risk: a 13% (1.13 [1.06–1.20]) increase below a −10 mm deviation in precipitation. Both light (<5 mm) and heavy runoff (≥5 mm) were associated with slightly statistically significant increases in rotavirus—respectively 11% (1.11 [1.01–1.22]) and 29% (1.29 [1.05–1.58])—while heavy runoff was associated with a moderately statistically significant increase in ETEC infection of 12% (1.12 [1.04–1.22]). Slight decreases in *Shigella* risk with light and heavy runoff were not statistically significant, though an equivalent decrease in *Campylobacter* risk with light runoff was.

Adjusted associations with solar radiation were inverse and low in magnitude for almost all pathogens and relative risks were not significant for any viruses, though *Cryptosporidium*, ETEC, *Giardia*, and *Shigella* did exhibit statistically significant reduced relative risks of infection above a 230 W/m^2^ threshold. Four of the five enteric viruses showed decreases in predicted prevalence at very low wind speeds, and for norovirus, this translated into a 13% decreased relative risk (0.87 [0.82–0.92]) following winds of below 2 m/s, though by contrast, relative risk of rotavirus increased by 13% (1.13 [1.05–1.21]) below the same threshold. Associations between wind speed and bacteria and protozoa were small in magnitude and mostly not statistically significant, although still conditions (<2 m/s winds) were associated with a statistically significant 8% (0.92 [0.89–0.95]) decrease in the relative risk of ETEC.

Predictions in Figures 4 and 5 are for the reference, asymptomatic stool sample category. For this reason, in several cases the adjusted probabilities are lower than the unadjusted probabilities, which are scaled to the positivity rates in the overall database with its large proportion of diarrheal samples. Multicollinearity could also cause this difference, but was found to be moderate and only between surface pressure and seasonality model terms (VIF < 7). Sensitivity analyses that excluded seasonality terms demonstrated much lower correlation between the hydrometeorological variables (VIF < 3) and only negligibly reduced differences between adjusted and unadjusted proportions. The results of all other sensitivity analyses (subsetting by sample type; excluding outlier sites such as Fortaleza; excluding the seasonality adjustment; and allowing more degrees of freedom in the spline terms) were consistent with those of the main analysis.

## 4 Discussion

The role of climatic drivers in the transmission of diarrheal disease remains relatively underexplored compared to some other disease groups and is challenging to characterize for numerous reasons. The etiology of the syndrome encompasses diverse microorganisms, which may be differentially sensitive to environmental conditions, yet clinically undifferentiated, with large reservoirs of asymptomatic transmission. Recent advances in broad spectrum molecular diagnostic technologies that can be deployed on stool samples from population‐based studies (Brown & Cumming, 2019; Lappan et al., 2021; Liu, Gratz, et al., 2016; Liu, Ochieng, et al., 2016), as well as climate data products that combine earth and ground‐based observations with model‐based reanalysis offer the potential to address these knowledge gaps (J. M. Colston, Ahmed, Mahopo, et al., 2018; J. M. Colston et al., 2019; Grace et al., 2015; M. C. Levy et al., 2019). This analysis is the first to bring together these two sources of data from multiple studies carried out in populations across diverse climate zones, resulting in a database of a scale and scope unprecedented in the study of enteropathogens and findings with implications for potential transmission pathways. A major strength of this analysis is that, by only including data from studies that tested for multiple pathogen targets in the same samples, we can have greater confidence in attributing observed effects to the exposures themselves rather than pathogen‐ and location‐specific factors. Furthermore, while health facility‐based studies offer logistical advantages such as minimizing the need for field work and transportation of samples, they may overestimate pathogen prevalence due to preferential recruitment of severely symptomatic diarrheal cases. By combining studies of both community‐ and health‐facility based designs, this study was able to adjust for these differences in the analysis stage, thus minimizing (though perhaps not fully eliminating) this bias.

Four main hypothesized mechanisms by which hydrometeorological conditions impact short‐term risk of enteropathogen transmission include waterborne dispersal (K. Levy et al., 2016), airborne dispersal (Dennehy, 2000; Ginn et al., 2021), survival on surfaces and fomites (Abad et al., 1994; Hurst et al., 1980) and host factors or behaviors influencing contact rates (T.‐C. Chan et al., 2015). In this context, “dispersal” can entail both the transport of infectious agents in a moving substrate—that is, precipitation runoff, or wind—or conversely their concentration or suspension during still conditions—in standing water or on infected aerosols in still air. These pathways are not clearly delineated, however, and these findings suggest that it may be possible for the same variable to impact different pathogens via different mechanisms and at varying magnitudes. Furthermore, hydrometeorological parameters are highly mutually interrelated. Surface pressure within a given season is generally interpreted as a proxy for storminess, for example, with lower‐than‐normal pressure more commonly associated with clouds (and correspondingly low solar radiation), rain, wind, and high pressure with clear skies and still conditions (Ahrens & Henson, 2018). High runoff indicates that precipitation has overwhelmed infiltration rates and occurs when very heavy rain falls over soil that is already close to fully saturated.

Regarding waterborne dispersal, several studies have reported evidence of a U‐shaped association between rainfall and enteric pathogen proliferation (Dunn & Johnson, 2018; Fang et al., 2019; Ikeda et al., 2019). A “concentration‐dilution” hypothesis has been proposed according to which microbes concentrate in water sources under very dry conditions, and are spread through the environment when heavy runoff or floodwater overwhelms drainage capacity (J. M. Colston et al., 2019; Kraay et al., 2020; K. Levy et al., 2016). Consistent with the first part of this theory, this analysis identified modest increases in risk for enteric viruses during periods when precipitation fell below the local average, with the exception of rotavirus which was, conversely, the only virus for which risk increased with heavier surface runoff. The only single pathogen that appeared to increase in prevalence with both low rainfall and heavy runoff was ETEC, a bacteria adapted to survive in riverine settings that is thought to have caused multiple diarrheal outbreaks in Dhaka, Bangladesh following severe flooding (Schwartz et al., 2006), though in this analysis, the effect sizes were small. This suggests that, if true, the concentration‐dilution hypothesis may not operate on any single pathogen, but rather by promoting proliferation of different etiological agents at either extreme of the precipitation distribution. An interrupted time series analysis of a large, La Niña‐related flood in the study site in Loreto, Peru found disruptions to the seasonal patterns of some of these pathogens over several months following the start of the flood, including increases in rotavirus and ETEC that are in line with the runoff findings identified here, but also increases in sapovirus, *Campylobacter*, *S* *higella*/EIEC and astrovirus, and decreases in adenovirus, which are at odds with them (J. Colston et al., 2020). That the effects of these two variables (precipitation and runoff) contrasted for different pathogens may relate to the differences in species‐specific incubation periods. Furthermore, the timing and sequence with which extremes of precipitation and runoff occur relative to the 7‐day window of aggregation used in this analysis may vary their effects on pathogens of different species and taxa. Rainwater from long periods of heavy precipitation may accumulate in subsequent and overlapping periods of heavy runoff, during which the environment remains saturated despite the rain itself having abated, promoting exposure to different pathogens at each stage of this process. It could also be an indication that whether prior baseline conditions are moist or arid could differentially influence the subsequent trajectory of a rainfall event's impact on pathogen dispersal (Kraay et al., 2020). This would implicate soil moisture as a variable on this pathway, since antecedent saturation levels of soil may determine the volume of rainfall that is converted to subsequent runoff with the potential to flush contaminants from the soil rather than infiltrating into the ground (D. Lee et al., 2019). It is notable that no associations were found between extreme rainfall or runoff and enteric protozoa risk, in contrast to the significant positive associations found in Canada between heavy precipitation and both cryptosporidiosis and giardiasis using much longer lags (4–6 weeks) than considered here (Chhetri et al., 2017). Taken together, these observations highlight the need to further examine impacts across longer lags and time windows and interactions between hydrological parameters that are specific to each individual pathogen, as well as to monitor extreme weather events such as ENSO‐related flooding.

Airborne transmission of enteropathogens is most commonly associated with the viruses, occurring via the suspension of aerosols from diaper disposal or toilet flushing or the formation of contaminated dry dust particles (Hervás et al., 2014; K. Levy et al., 2009). However, a recent study in urban sites in La Paz, Bolivia, and Kanpur, India was the first to test aerosol samples from near open wastewater canals using PCR and detected the presence of genes specific to enteropathogens of all three taxa included in this analysis, notably *Shigella*, ETEC, norovirus and *Cryptosporidium*, and suggesting a greater potential role for airborne transmission than was previously assumed (Ginn et al., 2021). Elsewhere, a study of diarrheal surveillance sites across China found that increased wind speed was followed by a reduction in the incidence of gastroenteritis attributable to enteric viruses (with the exception of the caliciviruses) and *Shigella* (L.‐P. Wang et al., 2021). This is in line with previous findings from analysis of rotavirus in MAL‐ED (J. M. Colston et al., 2019) and suggests that the suspension of infective aerosols in still air may be a mechanism for enteric virus transmission as it is for some respiratory and other airborne viruses (e.g., varicella zoster virus, measles, coronaviruses) (Leung, 2021; Tellier et al., 2019). The findings of this analysis largely conflict with this interpretation, however, since for all viruses except rotavirus, positivity was lowest in still conditions (Figure 4). The wind speed and surface pressure results reported here (the fifth and seventh columns of Figure 5) also suggest a stronger role for airborne transmission of bacteria and possibly protozoa than has previously been assumed (Gao et al., 2014; Hu et al., 2010; Nasr‐Azadani et al., 2017). Airborne zoonotic transmission of campylobacteriosis has been documented in poultry plant workers (Wilson, 2004) and prevalence of household‐scale poultry husbandry is high in many of the settings represented by these data (Schiaffino et al., 2020; Sultana et al., 2012). Similarly, airborne bioaerosols are a hypothesized secondary transmission mechanism for *E. coli* and *Cryptopsoridium* (W. L. Chan et al., 2019; Fujiyoshi et al., 2017; Sponseller et al., 2014). Though no such route has been considered for *Shigella*, and associations of this pathogen with wind speed were very modest, it is a similarly sized organism to *Campylobacter* (both <1.0 μm wide and <6.0 μm long) suggesting that transmission via air or houseflies acting as mechanical vectors may be equally plausible for the two species (Levine & Levine, 1991; Murray et al., 2013). At 8–12 μm in length, *Giardia duodenalis* cysts are the largest of the included infectious agents, which, though comparable in size to droplets involved in transmission of some respiratory infections (5–10 μm) (Murray et al., 2013; World Health Organization, 2014a) make it unlikely that the modest inverse association with wind speed identified here is causal. Furthermore, it is not clear how outdoor pressure and wind conditions might impact enteropathogen transmission among young children that largely occurs inside the dwelling. While indoor/outdoor pressure gradients influence the ventilation and air change rates of a dwelling in complex ways (Atkinson et al., 2009), if anything, this could be expected to lead to an inverse association between wind speed and infection due to stronger outside air movement increasing the evacuation of particles suspended in the air or on surfaces and fomites from indoors.

The strong associations of soil moisture, humidity and temperature with almost all pathogen taxa and species implicate these parameters as possible mediators influencing infection risk via pathogen survival outside the human host. Bacteria require moisture for their survival, which can be prolonged on surfaces on which large microdroplets have formed (Grinberg et al., 2019) and numerous viruses may remain viable for longer if they adhere to surfaces that permit retention of their surrounding moisture (Hervás et al., 2014; Kramer & Assadian, 2014). While several previous studies have speculated about how soil conditions might affect enteropathogen transmission (Aik et al., 2020; Gonzales‐Siles & Sjöling, 2016; Hasan et al., 2018; D. Lee et al., 2019; Rzezutka & Cook, 2004), few have attempted to quantify this effect (J. M. Colston et al., 2019). Almost all the pathogens, and most notably ETEC, exhibited peaks in risk at around 35°C, a value close to human body temperature. Previous studies have concluded that enteric virus transmission is associated with lower temperatures and humidity (K. Levy et al., 2016; Rzezutka & Cook, 2004; L.‐P. Wang et al., 2021). While this analysis detected inverse associations with humidity for all viruses except adenovirus (and particularly above a 65%–70% threshold), only rotavirus risk was found to decrease across the entire temperature range and to a marked degree, though adenovirus risk peaked at a lower temperature than most other pathogens (around 20°C). Contrastingly, astrovirus and the calicivirus actually showed slight increases in risk in the upper temperature range. Such countervailing effects of temperature on enteric viruses of different species may explain why a previous meta‐analysis did not find an association between ambient temperature and viral diarrheal disease incidence, despite identifying a strong, direct effect on bacterial and all‐cause diarrhea (Carlton et al., 2016). The modest associations of solar radiation with risk of several pathogens may be the direct influence of sunlight promoting or inhibiting microbial survival, or else the result of residual confounding of this variable with those on other pathways (such as rain clouds obscuring sunlight). *Giardia* and *Campylobacter* share a common zoonotic transmission pathway and household livestock may be important reservoirs of human infection for both pathogens (Daniels et al., 2016; St. Jean et al., 2021). However, given that *Giardia* is prone to infections of longer duration and with a lower attributable fraction of clinical diarrhea than *Campylobacter* (Platts‐Mills et al., 2018), the similarity in the unadjusted results between these two pathogens may partly be explained by subjects with *Campylobacter*‐attributable diarrhea being more likely to have a concurrent, persistent but asymptomatic *Giardia* infection.

This analysis is subject to several limitations. One is the inability to differentiate the impact of host‐mediated factors, such as susceptible individuals congregating indoors to escape extreme weather, thus increasing contact rates. This limitation is inherent to using data that were not collected with the specific objective of answering questions about climate, and would require different study designs to address, likely with intensive follow‐up and restricted to a small number of locations. A second limitation is the lack of consideration of interactions between exposures, distributed lags, and longer periods of exposure aggregation, effects that are likely to be highly relevant to pathogen transmission. For example, the effect of a heavy rainfall event may have opposing effects on pathogen dispersal depending on whether it follows a very dry period, causing the flushing of microbes into the environment, or a very wet period, leading to their dilution in runoff and standing water (Kraay et al., 2020; Lemaitre et al., 2019) or depending on the time period over which it is measured (J. Colston et al., 2020). For comparability across multiple pathogens it was necessary to limit the scope of this analysis to apply the exact same methods to all 10 infection outcomes, but future research can further explore the pathogen‐specific temporality behind and interactions between the identified associations. Other limitations include the inability to control for non‐climate factors such as drinking water, sanitation and healthcare access, which may be more proximal determinants of infection risk, and vary between countries and sites, but were beyond the scope of this study to explore. Finally, because some aspects of the analysis were not prespecified, the findings are sensitive to post hoc modeling choices, such as the choice of cutoff when dichotomizing continuous variables.

Climate change is bringing about shifts in the mean values for many of the parameters included in this analysis, as well as in their variability and frequency of extremes (Intergovernmental Panel on Climate Change [IPCC], 2012). Several studies have used projections in attempts to quantify how climate change might impact the burden of diarrheal disease (Kolstad & Johansson, 2011; World Health Organization, 2014b) or specific enteric pathogens (Chhetri et al., 2019), but found wide uncertainties around their estimates, perhaps due to only having considered single variables. Temperatures are rising almost everywhere (Intergovernmental Panel on Climate Change [IPCC] Working Group, 2013), and since all pathogens included in this study showed significant associations with that variable, these findings suggest that overall enteropathogen transmission is set to rise, with warmer locations experiencing increases in infections with bacterial and protozoal etiology, and cooler sites shifting into the mid‐range temperatures at which adenovirus and ETEC proliferate, but rotavirus transmission is suppressed. As the climate warms, both daily and seasonal precipitation variability will also increase over most land areas, but with dry areas getting drier on average (notably in southern Africa, Central America, and the Caribbean) and wet areas wetter (southern Asia and equatorial east Africa), translating into correspondingly more days with positive precipitation anomalies (Collins et al., 2013; Pendergrass et al., 2017). In combination with the results of this analysis, these predictions suggest that small increases in ETEC and viral enteric infections may be expected both in locations that are currently dry as they begin to experience even less daily rainfall, and in wetter areas as rainfall events become more extreme with correspondingly higher runoff volumes (Intergovernmental Panel on Climate Change [IPCC], 2012; Intergovernmental Panel on Climate Change [IPCC] Working Group, 2013). Decreases in the relative prevalence of bacterial and protozoal infections may also occur as daily precipitation and humidity shifts away from the mean and high runoff events become more frequent (Collins et al., 2013). However, accounting for general warming trends, changes in annual mean soil moisture follow the pattern of annual change in precipitation in most tropical and subtropical regions (Collins et al., 2013), so trends in waterborne bacteria and protozoa transmission may be compounded by increases in pathogen survival on moist soil and surfaces.

## 5 Conclusion

Different hydrometeorological variables have complex, often non‐linear associations with enteropathogen prevalence that vary in magnitude, direction and statistical significance depending on pathogen species and taxon and may engender shifts in the relative burden of different diarrhea‐causing agents as the global climate changes. Temperature and soil moisture are particularly influential parameters across all enteropathogen species, likely due to how they impact the survival of pathogens outside of the host, while hydrological factors (precipitation, surface runoff), as well as humidity have divergent effects both between enteric viruses and compared to most bacteria. Rising global temperatures may lead to decreases in adenovirus and rotavirus burden, and increases in the prevalence of enteric protozoa and bacteria, most notably ETEC, while the impacts of greater precipitation variability due to climate change on diarrhea‐causing pathogens are less certain and likely to be highly species‐ and location‐specific.

## Conflict of Interest

Drs. Gaensbauer and Melgar report grants from PanTheryx, Inc, and Dr. Page reports grants from GlaxoSmithKline, during the conduct of the study; the remaining authors have no conflicts of interests to disclose.

## Supporting information

Supporting Information S1

## Data Availability

The epidemiological data used in this analysis contains identifiable human subject data, which cannot be disseminated under the terms of the IRB and data use agreements with contributing institutions. Investigators from contributing studies may be contacted with reasonable request for data access. Data from the MAL‐ED and GEMS studies are available from the ClinEpiDB website (VEuPathDB, 2021). GLDAS data is disseminated as part of the mission of NASA's Earth Science Division and archived and distributed by the Goddard Earth Sciences (GES) Data and Information Services Center (DISC) (NASA, 2021).
