# Supplementary material for: Associations Between Eight Earth Observation‐Derived Climate Variables and Enteropathogen Infection: An Independent Participant Data Meta‐Analysis of Surveillance Studies With Broad Spectrum Nucleic Acid Diagnostics
Source: Geohealth. 2022 Jan 1;6(1):e2021GH000452. doi: 10.1029/2021GH000452 (PMC8729196; doi:10.1029/2021GH000452)
Supplement: Supplementary file 1 — Supporting Information S1 [file GH2-6-e2021GH000452-s001.pdf]

GeoHealth

Supporting Information for

Associations between 8 Earth Observation-derived climate variables and enteropathogen infection: An Independent Participant Data Meta-Analysis of surveillance studies with broad spectrum nucleic acid diagnostics

Josh M. Colston - Division of Infectious Diseases and International Health, University of Virginia School of Medicine, Charlottesville, VA, 22903, USA, josh.colston@virginia.edu
Benjamin F. Zaitchik - Department of Earth and Planetary Sciences, Johns Hopkins Krieger School of Arts and Sciences, Baltimore, 21218, MA, USA, zaitchik@jhu.edu
Hamada S. Badr - Department of Earth and Planetary Sciences, Johns Hopkins Krieger School of Arts and Sciences, Baltimore, 21218, MA, USA, badr@jhu.edu
Eleanor Burnett - Division of Viral Diseases, US Centers for Disease Control and Prevention, Atlanta, USA, wwg7@cdc.gov
Syed Asad Ali - Department of Pediatrics and Child Health, Aga Khan University, Karachi, 74800, Pakistan, asad.ali@aku.edu
Ajit Rayamajhi - Department of Pediatrics, National Academy of Medical Sciences, Kanti Children's Hospital, Kathmandu, Nepal, ajitrnp@yahoo.com
Syed M. Satter - Programme for Emerging Infections, Infectious Diseases Division, International Centre for Diarrhoeal Disease Research, Bangladesh (icddr,b), Dhaka, Bangladesh, dr.satter@icddrb.org
Daniel Eibach – Department of Infectious Disease Epidemiology, Bernhard Nocht Institute for Tropical Medicine (BNITM), Hamburg, Germany, eibach@bnitm.de
Ralf Krumkamp - Department of Infectious Disease Epidemiology, Bernhard Nocht Institute for Tropical Medicine (BNITM), Hamburg, Germany, krumkamp@bnitm.de
Jürgen May - Department of Infectious Disease Epidemiology, Bernhard Nocht Institute for Tropical Medicine (BNITM), Hamburg, Germany, may@bnitm.de
Roma Chilengi - Centre for Infectious Disease Research in Zambia, Lusaka, Zambia, roma.chilengi@cidrz.org
Leigh M. Howard - Department of Pediatrics, Vanderbilt University Medical Center, Nashville, TN, USA, leigh.howard@vumc.org
Samba O. Sow - Centre pour le Développement des Vaccins, Mali, Bamako, Mali, ssow@som.umaryland.edu
M. Jahangir Hossain - Medical Research Council Unit, The Gambia at the London School of Hygiene & Tropical Medicine, P. O. Box 273, Banjul, The Gambia, jhossain@mrc.gm
Debasish Saha - Epidemiology and Health Economics, GSK Vaccine, Wavre, 1300, Belgium, debasish.x.saha@gsk.com
M. Imran Nisar - Department of Pediatrics and Child Health, The Aga Khan University, Karachi, 74800, Pakistan, imran.nisar@aku.edu
Anita K. M. Zaidi - Department of Pediatrics and Child Health, The Aga Khan University, Karachi, 74800, Pakistan, anita.zaidi@aku.edu
Suman Kanungo - National Institute of Cholera and Enteric Diseases, Kolkota, India, sumankanungo@gmail.com
Inácio Mandomando - Centro de Investigação em Saúde de Manhiça, Manhiça, Mozambique, inacio.mandomando@manhica.net
Abu S. G. Faruque - Centre for Nutrition & Food Security, International Centre for Diarrhoeal Disease Research, Bangladesh (icddr,b), Dhaka, 1212, Bangladesh, gfaruque@icddrb.org

Karen L. Kotloff - Department of Pediatrics, University of Maryland School of Medicine, Baltimore, 21201, MD, USA, kkotloff@som.umaryland.edu
Myron M. Levine - Departments of Medicine and Pediatrics, Center for Vaccine Development and Global Health, University of Maryland School of Medicine, Baltimore, 21201, MD, USA, mlevine@som.umaryland.edu
Robert F. Breiman - Global Health, Rollins School of Public Health, Emory University, Atlanta, 30322, GA, USA, rfbreiman@emory.edu
Richard Omore - Kenya Medical Research Institute, Center for Global Health Research, Kisumu, Nyanza, 40100, Kenya, omorerichard@gmail.com
Nicola Page - Centre for Enteric Diseases, National Institute for Communicable Diseases, Pretoria, South Africa, nicolap@nicd.ac.za
James A. Platts-Mills - Division of Infectious Diseases and International Health, University of Virginia School of Medicine, Charlottesville, VA, 22903, USA, jp5t@virginia.edu
Ulla Ashorn - Center for Child, Adolescent and Maternal Health Research, Tampere University, Tampere, Finland, ulla.ashorn@tuni.fi
Yue-Mei Fan - Center for Child, Adolescent and Maternal Health Research, Tampere University, Tampere, Finland, yuemei.fan@tuni.fi
Prakash Sunder Shrestha - Department of Child Health, Institute of Medicine of Tribhuvan University, Kirtipur, 44618, Nepal, prakashsunder@hotmail.com
Tahmeed Ahmed - Nutrition and Clinical Services Division, International Centre for Diarrhoeal Disease Research, Bangladesh (icddr,b), Dhaka, 1212, Bangladesh, tahmeed@icddrb.org
Estomih Mduma - Haydom Global Health Institute, Haydom, P.O. Box 9000, Tanzania, estomduma@gmail.com
Pablo Penatero Yori - Division of Infectious Diseases and International Health, University of Virginia School of Medicine, Charlottesville, VA, 22903, USA, pyori@virginia.edu
Zulfiqar Bhutta - Department of Pediatrics and Child Health, Aga Khan University, Karachi, 74800, Pakistan, zulfiqar.bhutta@aku.edu
Pascal Bessong - HIV/AIDS & Global Health Research Programme, University of Venda, Thohoyandou, Limpopo, 0950, South Africa, Pascal.Bessong@univen.ac.za
Maribel P. Olortegui - Asociacion Benefica PRISMA, Iquitos, 16006, Peru, mparedeso@prisma.org.pe
Aldo A. M. Lima - Department of Physiology and Pharmacology, Faculty of Medicine, Federal University of Ceará, Fortaleza, 60020-181, Brazil, alima@ufc.br
Gagandeep Kang - Department of Gastrointestinal Sciences, Christian Medical College, Vellore, 632004, India, gkang@cmcvellore.ac.in
Jean Humphrey - Department of International Health, Johns Hopkins Bloomberg School of Public Health, Baltimore, 21205, MA, USA, jhumphr2@jhu.edu
Andrew J. Prendergast - Centre for Genomics and Child Health, Blizard Institute, Queen Mary University of London, London, E1 2AT, UK, a.prendergast@qmul.ac.uk
Robert Ntozini - Zvitambo Institute for Maternal and Child Health Research, Harare, Zimbabwe, r.ntozini@zvitambo.com
Kazuhisa Okada - Research Institute for Microbial Diseases, Osaka University, Osaka, Japan, kazuhisa@biken.osaka-u.ac.jp
Warawan Wongboot - Department of Medical Sciences, National Institute of Health, Nonthaburi, Thailand, warawan.w@dmsc.mail.go.th
James Gaensbauer - Center for Global Health, Department of Epidemiology, Colorado School of Public Health, Aurora, CO 80045. james.gaensbauer@dhha.org
Mario T. Melgar MD. Pediatric Infectious Diseases, Hospital Roosevelt, Guatemala City, Guatemala, CP 01011. mariomelgart@gmail.com
Tuula Pelkonen - Children's Hospital, Helsinki University Central Hospital, Helsinki, Finland, tuulapelkonen@hotmail.com
Cesar Mavacala Freitas - Hospital Pediátrico David Bernardino, Luanda, Angola, freitmav@yahoo.fr
Margaret N. Kosek - Division of Infectious Diseases and International Health and Public Health Sciences, University of Virginia School of Medicine, Charlottesville, VA, 22903, USA, mkosek@virginia.edu

Contents of this file

Model equation S1

Table S1: PCR targets used for each pathogen by each included study

Figure S1: Density plots of the distribution of the two hydrometeorological variables, which were recalculated as deviations from the location-specific mean in the contributing study sites, ordered by latitude from northernmost to southernmost.

Model equation S1:

$$\begin{aligned} Pr(Y_{ctsl} = Positive) &= \beta_0 + \beta_1 f(age_{ct}) + \beta_2 f(precipitation_{tl}) \\ &+ \beta_3 f(relativehumidity_{tl}) + \beta_3 f(soilmoisture_{tl}) \\ &+ \beta_4 f(solarradiation_{tl}) + \beta_5 f(surfacepressure_{tl}) \\ &+ \beta_6 f(temperature_{tl}) + \beta_7 f(windspeed_{tl}) + \beta_8 (surfacerunoff_{tl} \\ &= light) + \beta_9 (surfacerunoff_{tl} \\ &= heavy) \ + \beta_{10} i(diarrhea_{it}, design_s) + \beta_{11}(country_l) \\ &+ \beta_{12} i(zone_l, sine_t) + \beta_{13} i(zone_l, cosine_t) \end{aligned} \quad \text{(S1)}$$

where

$Pr(Y_{ctsl} = Positive)$ = The probability of a stool from child c enrolled in study s at location l being positive for pathogen Y on date t.

$f(v)$ = A series of spline terms for variable v

$i(v_1, v_2)$ = an interaction between variables v_1 and v_2

Age (age_{ct}) [months]

Precipitation ($precipitation_{tl}$) [mm]

Relative humidity ($relativehumidity_{tl}$) [%]

Soil moisture ($soilmoisture_{tl}$) [%]

Solar radiation ($solarradiation_{tl}$) [W/m2]

Surface pressure ($surfacepressure_{tl}$) [mbar]

Temperature ($temperature_{tl}$) [°C]

Wind speed ($windspeed_{tl}$) [m/s]

Surface runoff ($surfacerunoff_{tl}$=none 0mm|light <5mm|heavy ≥5mm)

Sample type ($diarrhea_{ct}$=asymptomatic|symptomatic) and study design (designs=community|health facility)

Country ($country_l$) [15 countries; India]

Seasonality ($zone_l$, $sine_t$; $zone_l$, $cosine_t$) [Southern Asia, Tropical Northern Hemisphere, Tropical Southern Hemisphere, Southern Africa]

Table S1: PCR targets used for each pathogen by each included study

		Adenovirus 40/41	Sapovirus	Astrovirus	Norovirus	Rotavirus	*Campylobacter* spp.	ETEC	*Shigella* spp./EIEC	*Cryptosporidium* spp.	*Giardia* spp.
1.	Agogo Presbyterian Hospital	-	-	Luminex xTAG® Gastrointestinal Pathogen Panel proprietary multiplex assays							
2.	Asian Intussusception Surveillance Network (AISN)	*Fiber gene*	*RdRp*	*Capsid*	*ORF1-2*	*NSP3*	*cpn60*	*LT, STh, STp*	*ipaH*	*18S*	*18S*
3.	Diarrhoeal Sentinel Surveillance Programme (DSSP)	-	*RdRp*	*Capsid*	*ORF1-2*	*NSP3*	*cpn60*	*LT, STh, STp*	*ipaH*	*18S*	*18S*
4.	Global Enteric Multicenter Study (GEMS)	*Fiber gene*	*RdRp*	*Capsid*	*ORF1-2*	*NSP3*	*cpn60*	*LT, STh, STp*	*ipaH*	*18S*	*18S*
5.	Haydom Lutheran Hospital	*Fiber gene*	*RdRp*	*Capsid*	*ORF1-2*	*NSP3*	*cpn60*	*LT, STh, STp*	*ipaH*	*18S*	*18S*
6.	iLiNS-DYAD	-	-	-	*ORF1*	*VP2*	*16S*	-	*ipaH*	*DNA J-like protein gene*	*Small subunit RNA*
7.	Malnutrition and Enteric Disease Study (MAL-ED)	*Fiber gene*	*RdRp*	*Capsid*	*ORF1-2*	*NSP3*	*cpn60*	*LT, STh, STp*	*ipaH*	*18S*	*18S*
8.	Novel Biomarkers of Environmental Enteropathy (NBEE)	*Fiber gene*	*RdRp*	*Capsid*	*ORF1-2*	*NSP3*	*cpn60*	*LT, STh, STp*	*ipaH*	*18S*	*18S*
9.	Programme for Awareness and Elimination of Diarrhoea (PAED)	-	-	Luminex xTAG® Gastrointestinal Pathogen Panel proprietary multiplex assays							
10.	Pediatric Hospital of Luanda	Mobidiag Amplidiag® proprietary multiplex assays									
11.	RECODISA	-	*RdRp*	*Capsid*	*ORF1-2*	*NSP3*	*cadF*	*eltA, eltB*	*ipaH*	*COWP*	*18S*
12.	Rotavac Trial, India	*Fiber gene*	*RdRp*	*Capsid*	*ORF1-2*	-	*cpn60*	*LT, STh, STp*	*ipaH*	*18S*	*18S*
13.	Sanitation Hygiene Infant Nutrition Efficacy (SHINE) Trial	*Fiber gene*	*RdRp*	*Capsid*	*ORF1-2*	*NSP3*	*cpn60*	*LT, STh, STp*	*ipaH*	*18S*	*18S*
14.	Thai Hospitals	*Hexon*	*capsid junction*	*ORF1a*	*ORF1-2*	*NSP3*	*gyrB*	*elt, est-h, est-p*	*ipaH*	*COWP*	*ITS1*
15.	Urban and Rural Guatemala	BioFire® FilmArray® Gastrointestinal (GI) Panel proprietary multiplex assays									

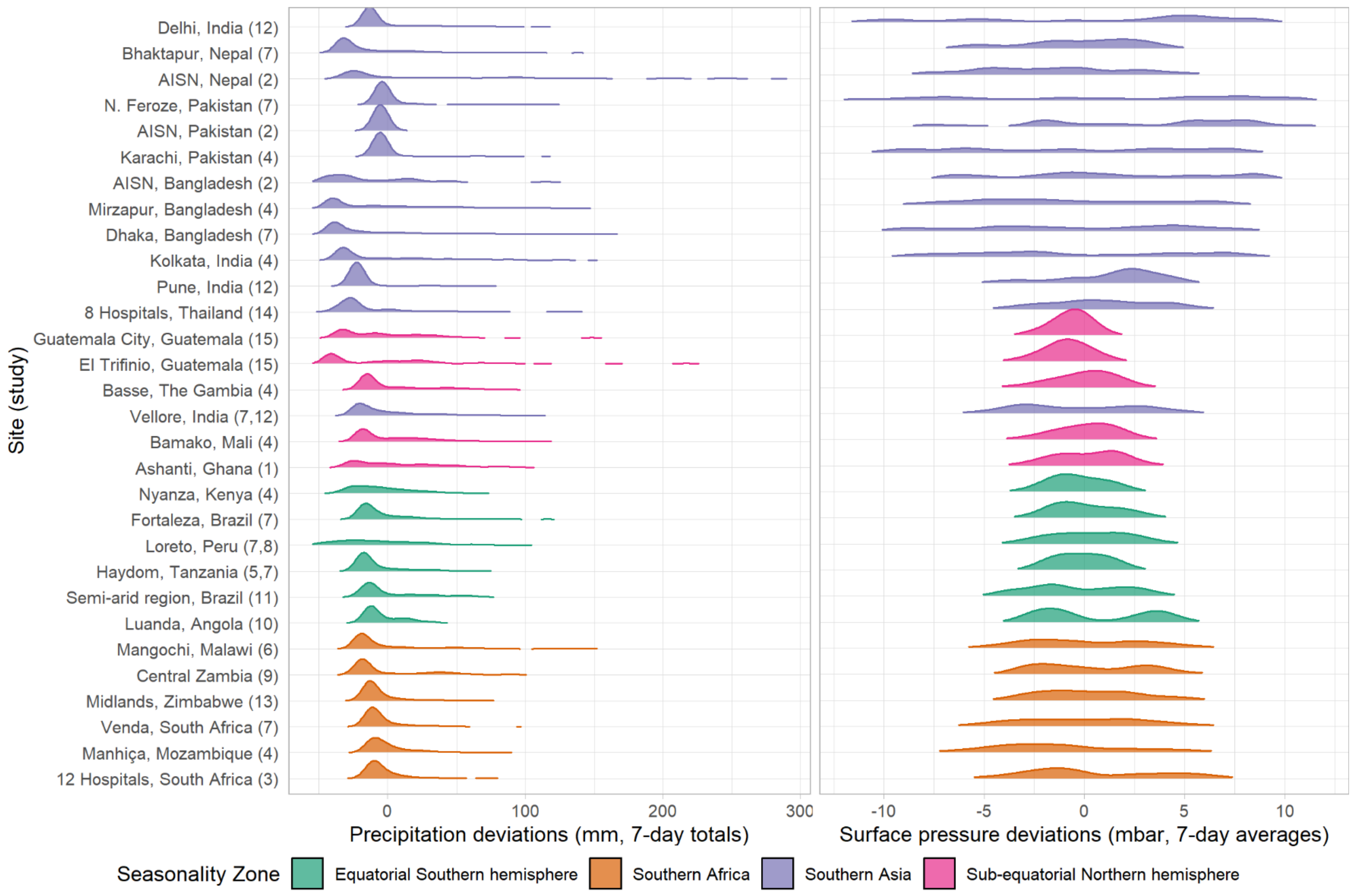

Figure **S1**: Density plots of the distribution of the two hydrometeorological variables, precipitation deviations (mm, 7-day totals) and surface pressure deviations (mbar, 7-day averages), which were recalculated as deviations from the location-specific mean in the contributing study sites, [with study sites] ordered by latitude from northernmost to southernmost."
